# Functional independence and spirometry in adult post-intensive care unit patients

**DOI:** 10.5935/0103-507X.20210031

**Published:** 2021

**Authors:** Lilian Regina Lengler Abentroth, Erica Fernanda Osaku, Mayara Manzoni Marques da Silva, Jaiane Luiza Jaskowiak, Renata de Souza Zaponi, Suely Mariko Ogasawara, Marcela Aparecida Leite, Cláudia Rejane Lima de Macedo Costa, Itamar Regazzo Pedreschi Porto, Amaury Cezar Jorge, Péricles Almeida Delfino Duarte

**Affiliations:** 1 General Adult Intensive Care Unit, Hospital Universitário do Oeste do Paraná, Universidade Estadual do Oeste do Paraná - Cascavel (PR), Brazil.

**Keywords:** Spirometry, Peak expiratory flow rate, Mobility limitation, Critical care, Respiration, artificial, Intensive care units, Ambulatory care facilities, hospital, Espirometria, Pico de fluxo expiratório, Limitação da mobilidade, Cuidados críticos, Respiração artificial, Unidades de terapia intensiva, Ambulatório hospitalar

## Abstract

**Objective:**

To relate functional independence to the degree of pulmonary impairment in adult patients 3 months after discharge from the intensive care unit.

**Methods:**

This was a retrospective cohort study conducted in one adult intensive care unit and a multi-professional post-intensive care unit outpatient clinic of a single center. Patients admitted to the intensive care unit from January 2012 to December 2013 who underwent (3 months later) spirometry and answered the Functional Independence Measure Questionnaire were included.

**Results:**

Patients were divided into groups according to the classification of functional independence and spirometry. The study included 197 patients who were divided into greater dependence (n = 4), lower dependence (n = 12) and independent (n = 181) groups. Comparing the three groups, regarding the classification of the Functional Independence Measure, patients with greater dependence had higher Acute Physiology and Chronic Health Evaluation II and Sequential Organ Failure Assessment values at intensive care unit admission with more advanced age, more days on mechanical ventilation, and longer stay in the intensive care unit and hospital. The majority of patients presented with pulmonary impairment, which was the obstructive pattern observed most frequently. When comparing functional independence with pulmonary function, it was observed that the lower the functional status, the worse the pulmonary function, with a significant difference being observed in peak expiratory flow (p = 0.030).

**Conclusion:**

The majority of patients who returned to the outpatient clinic 3 months after discharge had good functional status but did present with pulmonary impairment, which is related to the degree of functional dependence.

## Abstract (pt)

**Objetivo:**

Relatar a independência funcional e o grau de comprometimento pulmonar em pacientes adultos 3 meses após a alta da unidade de terapia intensiva.

**Métodos:**

Este foi um estudo de coorte retrospectiva conduzido em uma unidade de terapia intensiva multiprofissional para pacientes adultos em um único centro. Incluíram-se pacientes admitidos à unidade de terapia intensiva entre janeiro de 2012 e dezembro de 2013 que, 3 meses mais tarde, foram submetidos à espirometria e responderam ao questionário Medida de Independência Funcional.

**Resultados:**

Os pacientes foram divididos em grupos segundo sua classificação de independência funcional e espirometria. O estudo incluiu 197 pacientes, que foram divididos entre os grupos maior dependência (n = 4), menor dependência (n = 12) e independente (n = 181). Na comparação dos três grupos com relação à classificação pela Medida de Independência Funcional, pacientes com maior dependência tinham escores *Acute Physiology and Chronic Health Evaluation II e Sequential Organ Failure Assessment* mais altos quando da admissão à unidade de terapia intensiva, idade mais avançada, mais dias sob ventilação mecânica e tempo mais longo de permanência na unidade de terapia intensiva e no hospital. A maioria dos pacientes apresentava comprometimento pulmonar, sendo o padrão obstrutivo o mais frequentemente observado. Na comparação da independência funcional com a função pulmonar, observou-se que, quanto pior a condição funcional, pior a função pulmonar, observando-se diferenças significantes em relação ao pico de fluxo expiratório (p = 0,030).

**Conclusão:**

Em sua maioria, os pacientes que retornaram ao ambulatório 3 meses após a alta tinham boa condição funcional, porém apresentavam comprometimento pulmonar relacionado com o grau de dependência funcional.

## INTRODUCTION

In recent years, the number of patients who survive admission to the intensive care unit (ICU) has increased. It is estimated that 83% of ICU survivors have at least one readmission within 5 years, with 52% being readmitted in the first year, which generates a burden in regard to medical costs.^(1)^ In Brazil, the average daily cost of ICUs is high.^(2)^ Conversely, post-ICU morbidity and mortality are high among survivors, especially among those who require the greatest resources in the hospital.^(3)^ Most critical illness survivors require multi-professional care and long-term care services, generating a major public health problem due to long-term sequelae.^(4)^

Intensive care unit admission may lead to a number of complications and comorbidities, including muscle weakness due to immobility,^(5,6)^ physical limitations,^(6)^ pulmonary alterations,^(5)^ delirium^(7)^ and impairment of quality of life.^(8)^ This reduction in quality of life is generally associated with preexisting risk factors and poorer survival after hospital discharge.^(6,9)^ It has been observed that a fraction of patients present improvements in daily life activities six months after discharge from the ICU, but the return to work is affected.^(10,11)^ The use of prolonged mechanical ventilation (MV), neuromuscular blockers and length of ICU stay are factors that influence quality of life, functional independence and pulmonary function, even one year after a critical illness.^(12,13)^ Therefore, the evaluation of functional independence in patients after discharge is important to verify the effects of the hospitalization period, the consequences of immobility and the possibility of developing early interventions to minimize these complications.

In this way, scales have been used to evaluate the incapacities of patients who present functional restrictions and include items assessing motor and cognitive function and abilities to perform activities of daily living; these scales include the Functional Independence Measure (FIM), with which post-ICU patients with more than seven days of MV can be stratified into four groups of disability based on age and length of stay in the ICU, and the Barthel Index and the Sickness Impact Profile (SIP)^(14-17)^ Pulmonary complications after hospitalization in the ICU are related to respiratory failure, diaphragmatic weakness and acquired muscle weakness, which affect 25% of survivors, and spirometric alterations and abnormal lung volumes are also complications found in patients during long-term follow-up.^(18)^ Therefore, the assessment of pulmonary function is critical, as survivors of critical illness may present with changes in spirometric values within five years of ICU admission.^(19)^ It has been demonstrated that loss of mass and muscle strength, as well as the adjustment of MV, are factors that can generate pulmonary impairment post-ICU, contributing to decreased functional capacity that limits activities of daily living.^(18,20,21)^

Therefore, it is extremely important to assess the association between disorders of respiratory mechanics and changes in functional status, as impaired respiratory muscle function contributes to exercise intolerance, hinders return to work, compromises activities of daily living and significantly impacts the individual’s quality of life.

The objective of this study was to relate functional independence to the degree of lung function impairment in adult patients 3 months after discharge from the ICU, as well as to analyze the factors related to patient impairment.

## METHODS

The present study is a retrospective cohort study that evaluated factors associated with physical dependence and pulmonary function of patients admitted to an adult ICU with a Multiprofessional Post-ICU Outpatient Clinic (MPOC) at a university hospital in southern Brazil. The ICU was composed of 14 beds for trauma patients to provide clinical and surgical care. Data were collected from patients admitted to the ICU from January 2012 to December 2013, with a cross-sectional association between physical status and lung function.

### Study population and sample process

The study population consisted of patients who were discharged from the ICU and returned to the MPOC 3 months later for a multi-professional assessment, and the sampling was done for convenience. The study included patients who agreed to sign the Informed Consent form, who had cognitive and clinical conditions for performing spirometry and who answered the FIM questionnaire.

### Selected variables and collection method

The data were collected from the medical records, and the databank was related to the period of hospitalization in the ICU, hospital evolution and outpatient evaluation after 3 months of discharge from the ICU.

### Evaluation instruments (3 months after intensive care unit admission)

Pulmonary function was assessed by spirometry following the norms of the American Thoracic Society (ATS)^(22)^ and reference values ​​relative to the Brazilian population.^(23)^ The tests were performed by physical therapists using the One Flow FVC® portable spirometer (Clement Clarke, England) and met the criteria for acceptance and reproduction of maneuvers.

The FIM was used to evaluate the functional independence of patients and contains eighteen items encompassing six dimensions. Each item can receive scores of one to seven, where one corresponds to total dependency and seven to full independence.

Each dimension is analyzed by the sum of the items that compose it, and the total FIM score is given by the sum of each dimension, being able to vary from 18: complete dependence; 19 to 60: greater dependence (assistance of up to 50% of tasks); 61 to 103: less dependency (assistance of up to 25% of tasks); and 104 to 126: independent.^(14)^ For statistical purposes, the groups “complete dependence”, “greater dependence” and “less dependency” were eventually grouped as “dependent”.

### Statistical analysis

The data are described as the median and interquartile range. To verify the distribution of the data, the Kolmogorov-Smirnov test was used. Data were evaluated using Student’s t-test for independent samples when the assumption of normality was met. When nonnormally distributed, the data were evaluated using the Mann-Whitney test. Qualitative variables were assessed using the chi-square test for independence and analyzed based on absolute frequency. The association and relationship between variables were assessed using Pearson’s correlation. The statistical programs used were Statistical Package for Social Sciences (SPSS), XLSTat 2017 and R Core Team 2019, and p < 0.05 was considered statistically significant.

The present study was approved by the Research Ethics Committee of the *Universidade Estadual do Oeste do Paraná* under protocol 436.770 and conducted according to recommendations of resolution 466/2012 of the Brazilian National Health Council. Informed Consent Forms were signed by study participants or their close relatives.

## RESULTS

**Figure 1:**
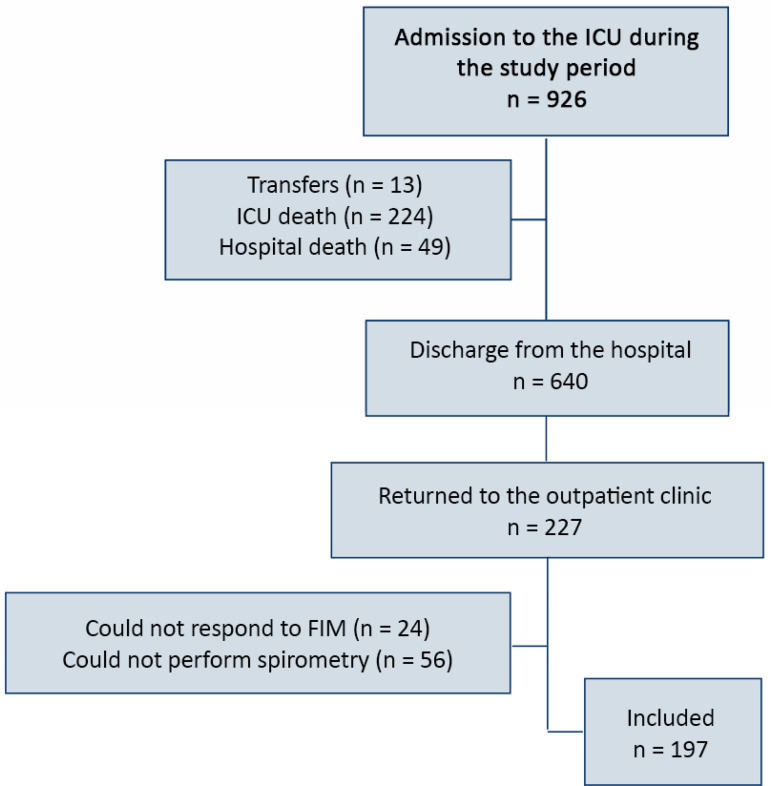
Flowchart of patients eligible for the study. ICU - intensive care unit; FIM - Functional Independence Measure.

In the study period, 697 patients were discharged alive from the ICU, of whom 277 (43%) attended the MPOC. Of these, 197 (71%) were eligible and included in the study (Figure 1).

Among the patients included in the study (n = 197), the main causes of admission were surgery (40%) and trauma (38%). The patients were predominantly male (62%), with a mean age of 42.1 years and admission Acute Physiology and Chronic Health Evaluation II (APACHE II) and Sequential Organ Failure Assessment (SOFA) scores of 19.4 and 8.1, respectively (Table 1S - Supplementary material). In addition, 37% of patients presented comorbidities prior to ICU admission, among which systemic arterial hypertension was the most prevalent (26% of patients).

**Table 1:** Characteristics of the sample according to functional dependence and independence as determined in the outpatient clinic (n = 197)

	Dependent	Independent	p value
	n = 16	n = 181	
Age (years)	54.5 (41.5 - 63.75)	39 (26 - 57)	< 0.0001
Male	10 (63)	112 (62)	0.9609
APACHE II	22.5 (15.75 - 26.25)	19 (13 - 24)	< 0.0001
SOFA (1st day ICU)	10 (7.75 - 11)	8 (5 - 11)	< 0.0001
Sedation (days)	38 (0 - 75.25)	10 (0 - 73)	< 0.0001
Mechanical ventilation > 24 hours	10 (63)	105 (58)	0.7270
Mechanical ventilation (hours)	132 (0 - 239.75)	41 (0 - 143)	< 0.0001
ICU stay (days)	9.5 (4.75 - 15)	5 (3 - 11)	< 0.0001
Hospital stay (days)	23 (18.25 - 25)	16 (10 - 23)	< 0.0001
Tracheostomy ICU	2 (13)	24 (13)	0.9314
Cause of admission			
Medical	4 (25)	40 (22)	0.7753
Trauma	7 (44)	68 (38)	
Surgical	5 (31)	73 (40)	
Comorbidities			
Hypertension	6 (38)	46 (25)	0.2931
Diabetes mellitus	3 (19)	16 (9)	0.1980
Neoplasm	2 (13)	18 (10)	0.7456
Congestive heart failure	-	6 (3)	-
No comorbidities	9 (56)	113 (62)	0.6255
Habits			
Smoker	8 (50)	80 (44)	0.6546
Pack/year	0 (0 - 0.25)	0 (0 - 4)	< 0.0001
Alcoholism	5 (31)	45 (25)	0.5736

APACHE II - Acute Physiology and Chronic Health Evaluation II; SOFA - Sequential Organ Failure Assessment; ICU - intensive care unit. Results expressed as median (interquartile range) or means of absolute frequency (%). A patient may present more than one comorbidity. Statistical significance (p < 0.05).

When patients were analyzed according to the FIM, it was verified that in the dependent group, the patients were older (p < 0.0001) and had greater clinical severity, APACHE II scores and SOFA scores (p < 0.0001). The duration of sedation, duration of MV, length of stay in the ICU and in the hospital were higher in patients with dependent function (p < 0.0001). The main cause of admission was trauma in the dependent group and surgery in the independent group (Table 1).

When evaluating lung function, 73% of patients had pulmonary impairment, and the obstructive pattern was the most predominant. A high prevalence of life habits such as smoking and alcoholism was found across the groups, especially in patients with normal and obstructive spirometry. Age was lower in the group with normal spirometry and higher in the obstructive group, and the predominant sex was male in the three groups (normal, obstructive and restrictive), presenting a significant difference in both variables and in the ventilation period greater than twenty-four hours (Table 2).

**Table 2:** Characterization of the sample according to spirometry

Variables	Spirometry %				p value
	Normal	Obstructive	Restrictive	Mixed	
	n = 54	n = 82	n = 53	n = 8	
Age (years)	30.5 (22 - 45)	48.5 (34 - 60.75)	36 (23 - 52)	39 (27.25 - 49.5)	0.005
Male	51 (94)	55 (67)	16 (30)	0	< 0.001
APACHE II	20 (16 - 23)	18.5 (12 - 24)	20 (11 - 24)	22 (19 - 28)	0.567
SOFA (1st day ICU)	9.5 (7 - 11)	8 (4.25 - 11)	6 (4 - 10)	10.5 (6 - 12)	0.076
Sedation (hours)	28 (0 - 96)	0 (0 - 48)	12 (0 - 60)	12 (0 - 72.25)	0.073
Mechanical ventilation (hours)	68 (24.5 - 153)	8.5 (0 - 144)	30 (0 - 172)	43.5 (6.75 - 89.75)	0.171
Mechanical ventilation > 24 hours	40 (74)	41 (50)	29 (54)	5 (62)	< 0.001
ICU stay (days)	6 (4 - 11)	5 (3 - 9.75)	5 (3 - 11)	8 (4.75 - 10.25)	0.389
Hospital stay (days)	17 (10.25 - 24)	16 (10.25 - 23.75)	18 (9 - 24)	18.5 (14.5 - 22.25)	0.965
Tracheostomy ICU	5 (9)	13 (15)	7 (13)	1 (12)	0.962
Comorbidities					
Hypertension	9 (16)	29 (35)	11 (20)	3 (37)	< 0.001
*Diabetes mellitus*	4 (7)	11 (13)	2 (3)	2 (25)	0.009
Neoplasm	11 (20)	10 (12)	9 (16)	0	0.158
*Congestive heart failure*	0	3 (3)	1 (1)	2 (25)	0.607
Chronic renal failure	0	4 (4)	0	1 (12)	0.180
No comorbidities	39 (72)	43 (52)	37 (69)	3 (37)	< 0.001
Habits					
Smoker	28 (51)	41 (50)	17 (32)	2 (25)	< 0.001
Packs/year	0 (0 - 4.75)	0 (0 - 7.5)	0	0	0.126
Alcoholism	18 (33)	25 (30)	7 (13)	0	0.007
Cause of admission					
Medical	10 (18)	23 (28)	7 (13)	4 (50)	< 0.001
Trauma	30 (55)	23 (28)	22 (41)	4 (50)	0.001
Surgical	14 (25)	36 (43)	24 (45)	0	< 0.001

APACHE II - Acute Physiology and Chronic Health Evaluation II; SOFA - Sequential Organ Failure Assessment; ICU - intensive care unit. Results presented as median (interquartile range) or means of absolute

**Figure 2:**
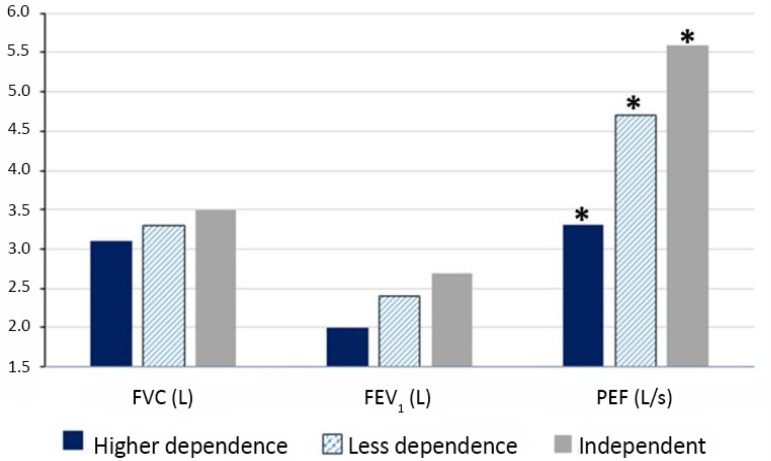
Comparison between functional independence measure classification and spirometric values. The units of the variables are different. FVC - forced vital capacity; FEV1- forced expiratory volume in the first second; PEF - peak expiratory flow. * p = 0.030 greater dependence versus less dependence versus independence.

When the FIM was compared with the spirometric values, it was observed that the group with the greatest dependence presented lower values of forced vital capacity (FVC), forced expiratory volume in the first second (FEV_1_) and peak expiratory flow (PEF) than the groups with greater independence (Figure 2).

For purposes of statistical correlation, patients with minor and major dependence were grouped together (dependent; n = 16) and compared with the independent group (n = 181). By Pearson’s correlation analysis, the correlation between respiratory variables (spirometric) and FIM values was weak for all parameters evaluated (Figure 1S - Supplementary material).

## DISCUSSION

In this study, pulmonary function and functional independence were evaluated in the MPOC 3 months after discharge from the ICU and were altered and showed a trend to associate with each other. The number of survivors of critical illness is increasing, and post-ICU care is evolving due to the emergence of new life-support techniques aimed at improving the quality of care provided to the individual.^(24)^ It is commonly found that post-ICU syndrome can generate physical, mental and cognitive alterations; therefore, multidisciplinary evaluation after discharge is of fundamental importance.^(25-27)^

Among the patients in this study, 37% presented previous diseases at hospital admission, with the most common being hypertension followed by diabetes mellitus. This result corroborates the findings described in the literature, which also found a predominance of these conditions in critical patients.^(27)^

Regarding the level of severity of the disease, as demonstrated by APACHE II and admission SOFA scores, it was observed that the scores did present a significant difference, and both were higher in the groups with functional dependence. In a study comparing individuals with and without sepsis, death rates among individuals were twice as high for up to 5 years in patients who had sepsis with higher APACHE II values and long-term sequelae, such as decreased quality of life and functional disability.^(28)^

Among the early and late consequences of physical and cognitive dysfunctions in patients after ICU, the decrease in functional capacity is reflected in physical/motor impairment with interference in activities of daily living. Critical status, need for MV and prolonged use of sedation may cause decreased functional independence and greater post-discharge disability, as has been observed in a number of post-ICU follow-up studies.^(12,13,15)^ In our study, the majority of patients achieved functional independence within 3 months after discharge from the ICU.

When patients in the study returned to the outpatient clinic, 92% already had near-normal functional status in all domains of the FIM questionnaire. This finding was consistent with a study that evaluated patients with brain injury and observed improvement in functional status after 6 weeks.^(29)^ In another similar study, improvements in functional independence were also found 6 months after discharge from the ICU,^(30)^ demonstrating that patients show long-term improvement in their daily life activities but noting that there are still important implications for the physical and pulmonary function of these patients.

This apparent contradiction could be explained by the age of our population, which is lower than the average in previous studies,^(31-33)^ primarily due to the high incidence of trauma and surgical admission causes. Therefore, we propose four possible reasons for most patients with good functional status: it is possible that a selection bias occurred, since many patients (57%) who were discharged from the hospital did not attend the outpatient clinic; patients who were included in the evaluation in contrast to those discharged from the hospital had less time on MV, sedation, fewer days in hospital and less tracheostomy; the time between discharge from the ICU and coming to the post-ICU outpatient clinic allowed recovery; and our study included a high incidence of young patients with trauma, who are more likely to have a rapid and effective recovery.

When comparing the functional status with the length of stay in the ICU, hospitalization time and time on MV, these factors were found to be higher in patients with more dependence, and significant differences were identified, corroborating the findings of a study that also made this comparison 6 months after discharge.^(30)^

Of the patients evaluated in the outpatient clinic, 73% had spirometric alterations, with the obstructive pattern being the most frequently observed in our population followed by the restrictive pattern. This result was also found in another study, where the author observed obstructive and restrictive spirometric changes in 25% of survivors of severe *acute respiratory distress syndrome (*ARDS) approximately 3 years after ICU discharge.^(34)^

When assessing lung function in ARDS survivors after 1 year, the restrictive pattern is reported to be the most common,^(35)^ which contrasts with the findings observed in our population. In another study, patients with ARDS were young with a long ICU stay and had normal spirometric measurements at 6 months of follow-up, but the carbon monoxide diffusion capacity remained low over the 12 months of follow-up.^(19)^ However, it is important to note that although the majority of our population was critically ill patients with a high incidence of MV, it was a heterogeneous population with different pulmonary conditions and was not limited to ARDS alone.

An important factor in our study that can justify the spirometric findings is the high incidence of smoking in our population. The same was associated with the length of hospital stay, and the use of prolonged MV may have influenced lung function in the long term. The fact that there is no baseline spirometric evaluation makes it impossible to state that such changes are due to previous conditions, secondary to acute illness or admission to the ICU. This gap is not specific to this study, with the exception of studies evaluating patients with elective surgeries or with previous respiratory diseases, but from most studies that assess the lung function of critical survivors.

In our population, diabetes was among the most prevalent comorbidities. One previous study demonstrated a significant association between type II diabetes and reduced pulmonary function with a decrease in FVC and FEV_1_.^(36)^ Diabetes has been associated with an increased risk for developing pneumonia, chronic obstructive pulmonary disease, asthma, and pulmonary fibrosis.^(37)^

We found in this study that patients with greater functional dependence presented lower spirometric values than those of individuals in the other groups. In patients submitted to extensive abdominal surgery, the evaluation of spirometry and FIM performed 30 days after surgical intervention obtained values ​​close to normal, and demonstrated that most patients were independent.^(38)^ A similar study evaluating preoperative abdominal surgery patients demonstrated that PEF and degree of functional independence were correlated with each other.^(39)^

When comparing spirometry with functional status, PEF has been shown to be significantly higher in patients with preserved functional independence. The majority of patients in our study presented with alterations in pulmonary function, and it was observed that, in general, the better the degree of functional independence is, the better the values ​​of FVC, FEV_1_ and PEF are.

Several limitations are found in the present study, which may eventually restrict the applicability and generalizability of its results. These limitations included the collection of data in a single center, with the outpatient evaluation being performed once 3 months after discharge from the unit. The study was conducted with a preselected population, and the excluded individuals were those who were unable to perform spirometry or respond to the FIM questionnaire. Despite being a cohort of patients with a well-established follow-up, the main analysis of the study was cross-sectional, with possible biases (e.g., reverse causality). For example, pulmonary diseases prior to ICU admission were not described or measured because of the difficulty of characterizing them, and the only clinical evaluation and respiratory complications resulting from hospitalization were not classified; for this reason, we acknowledge this limitation. The patient population in our study did not present significant prior sequelae; it also had specific characteristics, including a high incidence of trauma and young age, and may not correspond to the populations of other ICUs or social realities. The comparison with other spirometric findings was limited in our study because it was a heterogeneous population. In addition, there were losses in post-ICU follow-up due to difficulty in transportation and locomotion to the outpatient clinic due to the socioeconomic status of the patients. However, we emphasize the importance of spirometric performance in the detection of post-ICU respiratory disorders, smoking cessation in this group of patients, and the assessment of functionality, allowing adequate monitoring and rehabilitation.

## CONCLUSION

In a sample of patients analyzed 3 months after discharge from the intensive care unit, most of the survivors had a decline in lung function. The compromised lung function was related to the degree of functional dependence, primarily in relation to peak expiratory flow and forced expiratory volume in the first second.

## References

[1] Mikkelsen ME (2017). Triangulating weakness, morbidity, and mortality among acute respiratory distress syndrome survivors: a story emerges. Crit Care Med.

[2] Sogayar AM, Machado FR, Rea-Neto A, Dornas A, Grion CM, Lobo SM, Tura BR, Silva CL, Cal RG, Beer I, Michels V, Safi J, Kayath M, Silva E, Costs Study Group - Latin American Sepsis Institute (2008). A multicentre, prospective study to evaluate costs of septic patients in Brazilian intensive care units. Pharmacoeconomics.

[3] Hill AD, Fowler RA, Pinto R, Herridge MS, Cuthbertson BH, Scales DC (2016). Long-term outcomes and healthcare utilization following critical illness--a population-based study. Crit Care.

[4] Koster-Brouwer M, van de Groep K, Klein Klouwenberg P, Pasma W, van der Poll T, Bronten M (2015). Ongoing health care expenditure in survivors of sepsis in the intensive care unit. Intensive Care Med Exp.

[5] Kramer CL (2017). Intensive care unit-acquired weakness. Neurol Clin.

[6] Dinglas VD, Aronson Friedman L, Colantuoni E, Mendez-Tellez PA, Shanholtz CB, Ciesla ND (2017). Muscle weakness and 5-year survival in acute respiratory distress syndrome survivors. Crit Care Med.

[7] Bashar FR, Vahedian-Azimi A, Hajiesmaeili M, Salesi M, Farzanegan B, Shojaei S, Goharani R, Madani SJ, Moghaddam KG, Hatamian S, Moghaddam HJ, Mosavinasab SMM, Elamin EM, Miller AC, MORZAK Collaborative (2018). Post-ICU psychological morbidity in very long ICU stay patients with ARDS and delirium. J Crit Care.

[8] Hermans G, Van den Berghe G (2015). Clinical review: intensive care unit acquired weakness. Crit Care.

[9] Wieske L, Dettling-Ihnenfeldt DS, Verhamme C, Nollet F, van Schaik IN, Schultz MJ (2015). Impact of ICU-acquired weakness on post-ICU physical functioning: a follow-up study. Crit Care.

[10] Bigatello LM, Stelfox HT, Berra L, Schmidt U, Gettings EM (2007). Outcome of patients undergoing prolonged mechanical ventilation after critical illness. Crit Care Med.

[11] Hodgson CL, Udy AA, Bailey M, Barrett J, Bellomo R, Bucknall T (2017). The impact of disability in survivors of critical illness. Intensive Care Med.

[12] Damuth E, Mitchell JA, Bartock JL, Roberts BW, Trzeciak S (2015). Long-term survival of critically ill patients treated with prolonged mechanical ventilation: a systematic review and meta-analysis. Lancet Respir Med.

[13] Dettmer MR, Damuth E, Zarbiv S, Mitchell JA, Bartock JL, Trzeciak S (2017). Prognostic factors for long-term mortality in critically ill patients treated with prolonged mechanical ventilation a systematic review. Crit Care Med.

[14] Granger CV, Hamilton BB, Keith RA, Zielezny M, Shersin FS (1986). Advances in functional assessment for rehabilitation. Top Geriatr Rehabil.

[15] Curzel J, Forgiarini Junior LA, Rieder MM (2013). Evaluation of functional independence after discharge from the intensive care unit. Rev Bras Ter Intensiva.

[16] Jesus FS, Paim DM, Brito JO, Barros IA, Nogueira TB, Martinez BP (2016). Mobility decline in patients hospitalized in an intensive care unit. Rev Bras Ter Intensiva.

[17] Herridge MS, Chu LM, Matte A, Tomlinson G, Chan L, Thomas C, Friedrich JO, Mehta S, Lamontagne F, Levasseur M, Ferguson ND, Adhikari NK, Rudkowski JC, Meggison H, Skrobik Y, Flannery J, Bayley M, Batt J, Santos CD, Abbey SE, Tan A, Lo V, Mathur S, Parotto M, Morris D, Flockhart L, Fan E, Lee CM, Wilcox ME, Ayas N, Choong K, Fowler R, Scales DC, Sinuff T, Cuthbertson BH, Rose L, Robles P, Burns S, Cypel M, Singer L, Chaparro C, Chow CW, Keshavjee S, Brochard L, Hebert P, Slutsky AS, Marshall JC, Cook D, Cameron JI, RECOVER Program InvestigatorsCanadian Critical Care Trials Group (2016). The RECOVER Program: disability risk groups and 1-year outcome after 7 or more days of mechanical ventilation. Am J Respir Crit Care Med.

[18] Damm T, Patel JJ (2015). Long-term outcomes after critical illness: a concise clinical review. PulmCCM J.

[19] Herridge MS, Tansey CM, Matté A, Tomlinson G, Diaz-Granados N, Cooper A, Guest CB, Mazer CD, Mehta S, Stewart TE, Kudlow P, Cook D, Slutsky AS, Cheung AM, Canadian Critical Care Trials Group (2011). Functional disability 5 years after acute respiratory distress syndrome. N Engl J Med.

[20] Sillanpää E, Stenroth L, Bijlsma AY, Rantanen T, McPhee JS, Maden-Wilkinson TM (2014). Associations between muscle strength, spirometric pulmonary function and mobility in healthy older adults. Age (Dordr).

[21] Toufen Junior C, De Santis Santiago RR, Hirota AS, Carvalho AR, Gomes S, Amato MB (2018). Driving pressure and long-term outcomes in moderate/severe acute respiratory distress syndrome. Ann Intensive Care.

[22] Miller MR, Hankinson J, Brusasco V, Burgos F, Casaburi R, Coates A, Crapo R, Enright P, van der Grinten CP, Gustafsson P, Jensen R, Johnson DC, MacIntyre N, McKay R, Navajas D, Pedersen OF, Pellegrino R, Viegi G, Wanger J, ATS/ERS Task Force (2005). Standardisation of spirometry. Eur Respir J.

[23] Pereira CA, Sato T, Rodrigues SC (2007). New reference values for forced spirometry in white adults in Brazil. J Bras Pneumol.

[24] Battle CE, James K, Bromfield T, Temblett P (2017). Predictors of post-traumatic stress disorder following critical illness: a mixed methods study. J Intensive Care Soc.

[25] Lone NI, Gillies MA, Haddow C, Dobbie R, Rowan KM, Wild SH (2016). Five-year mortality and hospital costs associated with surviving intensive care. Am J Respir Crit Care Med.

[26] Eakin MN, Patel Y, Mendez-Tellez P, Dinglas VD, Needham DM, Turnbull AE (2017). Patients' outcomes after acute respiratory failure: a qualitative study with the PROMIS framework. Am J Crit Care.

[27] Varghese YE, Kalaiselvan MS, Renuka MK, Arunkumar AS (2017). Comparison of acute physiology and chronic health evaluation II (APACHE II) and acute physiology and chronic health evaluation IV (APACHE IV) severity of illness scoring systems, in a multidisciplinary ICU. J Anaesthesiol Clin Pharmacol.

[28] Wang HE, Szychowski JM, Griffin R, Safford MM, Shapiro NI, Howard G (2014). Long-term mortality after community acquired sepsis: a longitudinal population-based cohort study. BMJ Open.

[29] Oujamaa L, Francony G, Boucheix P, Schilte C, Bouzat P, Perennou D (2017). Dynamics of clinical recovery during the early phase of rehabilitation in patients with severe traumatic and non-traumatic brain injury. Brain Inj.

[30] Dennis DM, Hebden-Todd TK, Marsh LJ, Cipriano LJ, Parsons RW (2011). How do Australian ICU survivors fare functionally 6 months after admission. Crit Care Resusc.

[31] Casamento A, Bailey M, Robbins R, Pilcher D, Warrillow S, Ghosh A (2018). Patient characteristics, incidence, technique, outcomes and early prediction of tracheostomy in the state of Victoria, Australia. J Crit Care.

[32] Gantner D, Farley K, Bailey M, Huckson S, Hicks P, Pilcher D (2014). Mortality related to after-hours discharge from intensive care in Australia and New Zealand, 2005-2012. Intensive Care Med.

[33] Demircan A, Aygencel Bikmaz SG, Kadi G, Keles A, Bildik F, Öktem B (2017). Evaluation of the general characteristics of patients aged 85 years and above admitted to a university hospital emergency department. Turk J Med Sci.

[34] Neff TA, Stocker R, Frey HR, Stein S, Russi EW (2003). Long-term assessment of lung function in survivors of severe ARDS. Chest.

[35] Wang ZY, Li T, Wang CT, Xu L, Gao XJ (2017). Assessment of 1-year outcomes in survivors of severe acute respiratory distress syndrome receiving extracorporeal membrane oxygenation or mechanical ventilation: a prospective observational study. Chin Med J (Engl).

[36] Ehrlich SF, Quesenberry CP Jr, Van Den Eeden SK, Shan J, Ferrara A (2010). Patients diagnosed with diabetes are at increased risk for asthma, chronic obstructive pulmonary disease, pulmonary fibrosis, and pneumonia but not lung cancer. Diabetes Care.

[37] Klein OL, Kalhan R, Williams MV, Tipping M, Lee J, Peng J (2012). Lung spirometry parameters and diffusion capacity are decreased in patients with Type 2 diabetes. Diabet Med.

[38] Soares SM, Nucci LB, da Silva MM, Campacci TC (2013). Pulmonary function and physical performance outcomes with preoperative physical therapy in upper abdominal surgery: a randomized controlled trial. Clin Rehabil.

[39] Czyzewski P, Szczepkowski M, Domaniecki J, Dabek A (2013). Physiotherapy based on PNF concept for elderly people after conventional colon surgery. Pol Przegl Chir.

