# Supplementary material for: Functional independence and spirometry in adult post-intensive care unit patients
Source: Rev Bras Ter Intensiva. 2021 Apr-Jun;33(2):243–50. doi: 10.5935/0103-507X.20210031 (PMC8275088; doi:10.5935/0103-507X.20210031)
Supplement: Supplementary file 1 [file rbti-33-02-0243-suppl01.pdf]

Functional independence and spirometry in adult post-intensive care unit patients

Independência funcional e espirometria em pacientes adultos pós-unidade de terapia intensiva

Lilian Regina Lengler Abentroth[1], Erica Fernanda Osaku[1], Mayara Manzoni Marques da Silva[1], Jaiane Luiza Jaskowiak[1], Renata de Souza Zaponi[1], Suely Mariko Ogasawara[1], Marcela Aparecida Leite[1], Cláudia Rejane Lima de Macedo Costa[1], Itamar Regazzo Pedreschi Porto[1], Amaury Cezar Jorge[1], Péricles Almeida Delfino Duarte[1]

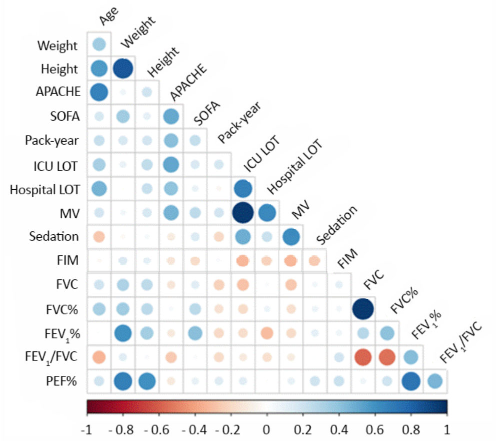

Figure 1S - Pearson's correlation analysis between respiratory variables and Functional Independence Measure values.
APACHE II - Acute Physiology and Chronic Health Evaluation II; SOFA - Sequential Organ Failure Assessment; ICU - intensive care unit; MV - mechanical ventilation; FIM - Functional Independence Measure; FVC - forced vital capacity; FEV1 - forced expiratory volume in the first second; PEF - peak expiratory flow.

Table 1S - General characteristics of patients who were discharged from the hospital and those included in the study

Variables	Characteristics of patients		p value
	Discharged from hospital	Included in the study	
	n = 640	n = 197	
Age (years)	44.8 ± 19.53	42.1 ± 18.27	0.001
Male sex	63	62	0.865
APACHE II	19.7 ± 7.70	19.4 ± 7.50	0.636
Sedation (days)	3.4 ± 5.22	2.4 ± 4.28	0.014
Mechanical ventilation (days)	6.2 ± 8.39	4.3 ± 5.93	0.003
ICU stay (days)	9.8 ± 9.08	8.0 ± 6.76	0.010
Hospital stay (days)	25.7 ± 21.34	19.2 ± 13.07	< 0.001
Mechanical ventilation > 24 hours	62	58	0.356
Tracheostomy ICU	23	13	0.003
Cause of admission			
Medical	30	22	0.036
Trauma	38	38	
Surgical	32	40	
Comorbidities			
Hypertension	23	26	0.441
Diabetes mellitus	8	10	0.462
Neoplasm	14	10	0.182
Congestive heart failure	6	3	0.144
Chronic renal failure	2	3	0.579
No comorbidities	60	63	0.503
Habits			
Smoker	13	45	< 0.001
Alcoholism	14	25	< 0.001
Spirometry in outpatient clinic			
Normal	-	27	-
Obstructive	-	42	-
Restrictive	-	27	-
Mixed	-	4	-

APACHE II - Acute Physiology and Chronic Health Evaluation II; ICU - intensive care unit. Results presented as mean ± standard deviation or %. A patient may present more than one prior disease (comorbidities).
